# First Biomimetic Fixation for Resurfacing Arthroplasty: Investigation in Swine of a Prototype Partial Knee Endoprosthesis

**DOI:** 10.1155/2019/6952649

**Published:** 2019-07-01

**Authors:** Piotr Rogala, Ryszard Uklejewski, Mariusz Winiecki, Mikołaj Dąbrowski, Jacek Gołańczyk, Adam Patalas

**Affiliations:** ^1^Department of Orthopaedics and Traumatology, W. Dega University Hospital, Poznan University of Medical Sciences, 28 Czerwca 1956 Street 135/147, 61-545 Poznan, Poland; ^2^Institute of Health Sciences, Hipolit Cegielski State College of Higher Education, Card. Stefan Wyszyński Street 38, 62-200 Gniezno, Poland; ^3^Veterinary Surgery Department, Naramowicka Street 68, 61-619 Poznań, Poland; ^4^Department of Medical Bioengineering Fundamentals, Institute of Technology, Kazimierz Wielki University, Karol Chodkiewicz Street 30, 85-064 Bydgoszcz, Poland; ^5^Department of Technology Design/Laboratory of Bone Implants Research and Design, Institute of Mechanical Technology, Poznan University of Technology, Piotrowo Street 3, 60-965 Poznan, Poland; ^6^Department of Spondyloorthopaedics and Biomechanics, W. Dega University Hospital, Poznan University of Medical Sciences, 28 Czerwca 1956 Street 135/147, 61-545 Poznan, Poland

## Abstract

Resurfacing hip and knee endoprostheses are generally embedded in shallow, prepared areas in the bone and secured with cement. Massive cement penetration into periarticular bone, although it provides sufficient primary fixation, leads to the progressive weakening of peri-implant bone and results in failures. The aim of this paper was to investigate in an animal model the first biomimetic fixation of components of resurfacing arthroplasty endoprostheses by means of the innovative multispiked connecting scaffold (MSC-Scaffold). The partial resurfacing knee arthroplasty (RKA) endoprosthesis working prototype with the MSC-Scaffold was designed for biomimetic fixation investigations using reverse engineering methods and manufactured by selective laser melting. After Ca-P surface modification of bone contacting surfaces of the MSC-Scaffold, the working prototypes were implanted in 10 swines. Radiological, histopathological, and micro-CT examinations were performed on retrieved bone-implant specimens. Clinical examination confirmed very good stability (4 in 5-point Likert scale) of the operated knee joints. Radiological examinations showed good implant fixation (radiolucency less than 2 mm) without any signs of migration. Spaces between the MSC-Scaffold spikes were penetrated by bone tissue. The histological sections showed newly formed trabecular bone tissue between the spikes, and the trabeculae of periscaffold bone were seen in contact with the spikes. The micro-CT results showed the highest percentage of bone tissue ingrowths into the MSC-Scaffold at a distance of 2.5÷3.0 mm from the spikes bases. The first biomimetic fixation for resurfacing arthroplasty was successfully verified in 10 swines investigations using RKA endoprosthesis working prototypes. The performed research shows that the MSC-Scaffold allows for cementless and biomimetic fixation of resurfacing endoprosthesis components in periarticular cancellous bone.

## 1. Introduction

Contemporary resurfacing hip and knee endoprostheses are generally embedded in shallow prepared areas in the bone and secured with cement. In the case of hip resurfacing arthroplasty (RA) endoprostheses, a hybrid fixation technique using a cemented short-stem femoral component in combination with an uncemented acetabular component is used. The observed interface and periprosthetic necrosis is frequently followed by loosening, resulting in migration of the femoral component or femoral neck fractures. Consequently, due to these major postoperative complications [1–9], primary hip RA revision is unavoidable, in which femoral-only revision occurs in 62% of all cases [10].

In the case of partial knee RA endoprostheses, the components are commonly secured with cement and drill holes are made in the femoral condyle to accommodate the two pegs used to stabilize the implant. Extensive cement penetration into the periprosthetic bone causes remodeling and resorption of the bone surrounding the cement [11]. The appearance of peripheral radiolucent lines (RLLs) under the component, defined as intervals between the cement and the bone, is a frequent occurrence and is associated with the preparation of the bone surface [12]. In cases where pain is accompanied by RLLs, this can be considered a cause of loosening and may lead to unnecessary revisions [13, 14]. The use of uncemented knee resurfacing has been trialed for some time and has shown the potential to improve bone-component fixation while removing problems associated with cement, such as foreign body retention [15, 16]. Cementless fixation is associated with fewer RLLs and no increase in complication rates [14].

In this paper, the essential innovation in the fixation of components of joint RA endoprostheses in the periarticular trabecular bone is presented, the biomimetic fixation by means of the multispiked connecting scaffold (MSC-Scaffold), interfacing the resurfacing endoprosthesis components with bone. This biomimetic fixation was designed, manufactured, and tested by Uklejewski et al. [17–19], based on the concept of needle-palisade bone fixation developed by Rogala [20–22]. The MSC-Scaffold's spikes mimic the interdigitations of articular subchondral bone, which is the natural biostructure interfacing the articular cartilage with the periarticular trabecular bone. Against many observed failures of standard cemented fixation of contemporary total hip RA endoprostheses, where the femoral component is always fixed with the use of cement, the proposed biomimetic fixation can be regarded a promising breakthrough in advanced bone-implant interfacing in joint RA endoprostheses. This biomimetic fixation manufactured by selective laser melting (SLM) represents a new generation of biomimetic RA endoprostheses, which can be applied for the most diarthrodial joint arthroplasties used in orthopaedic surgery treatment.

**Figure 1:**
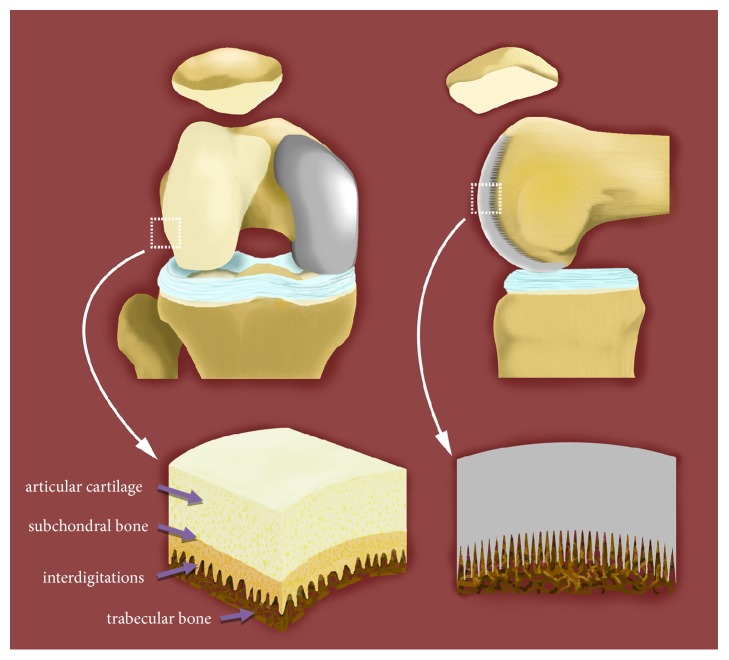
Diagram of articular hyaline cartilage and subchondral bone with interdigitations interlocking with trabeculae of cancellous bone vs. the first biomimetic fixation of components of resurfacing arthroplasty endoprostheses by means of the multispiked connecting scaffold (MSC-Scaffold).

Figure 1 provides a diagram of the knee joint, showing articular hyaline cartilage and subchondral bone with interdigitations interlocking with the trabeculae of cancellous bone vs. the first biomimetic fixation of the components of RA endoprostheses by means of the MSC-Scaffold.

Recent investigations on the MSC-Scaffold prototype have included, among others, the examination of possibilities of modifying the interspike structural proosteoconductive potential of the SLM-manufactured MSC-Scaffold [23]. The most influential geometrical features of the MSC-Scaffold to ensure the closest-to-physiological load transfer in the peri-implant bone were determined in numerical simulations [24]. The initial pilot implantation study in an animal model of the structurally functionalized MSC-Scaffold [25] provided promising results; the scaffolding effect was obtained with the MSC-Scaffold preprototypes, and the majority of the interspike pore space was penetrated with newly formed bone tissue, providing primary biological fixation of the MSC-Scaffold preprototypes in the periarticular cancellous bone. It was concluded that improved osseointegration on the MSC-Scaffold surface should be sought, using calcium phosphates or hydroxyapatite [25]. To enhance the osteoinduction/osseointegration potential of the MSC-Scaffold prototype, the research to modify its bone contacting surfaces via the process of electrochemical cathodic deposition was carried out at constant current densities with further immersion of the MSC-Scaffold preprototypes in simulated body fluid in order to transform the amorphous calcium phosphate (Ca-P) coating into a hydroxyapatite-like coating. The results demonstrating the effect of the enhancement of the osteoinduction/osseointegration potential of the Ca-P modified MSC-Scaffold preprototypes in an experimental study using animal models (acc. ISO 10993) and an osteoblast cell culture evaluation were presented and discussed in previous work by Uklejewski al. [26].

The aim of this paper was to investigate in an animal model the first biomimetic fixation of components of RA endoprostheses by means of the innovative MSC-Scaffold. For this purpose, the partial resurfacing knee arthroplasty (RKA) endoprosthesis working prototype with the MSC-Scaffold was designed for swine using reverse engineering methods and manufactured by selective laser melting.

## 2. Materials and Methods

The swine femur (breed: Polish Large White) was taken from a nine-month-old boar (weight 87 kg) and used to create the 3D virtual model of the partial RKA endoprosthesis working prototype with the MSC-Scaffold. The bone was acquired from a local slaughterhouse and then mechanically cleaned of all soft tissues and fixed in 10% formalin solution for one week.

**Figure 2:**
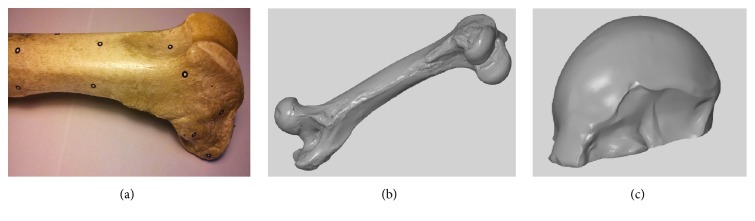
The main steps of 3D bone reconstruction: (a) swine femoral bone prepared for 3D scanning with reference markers attached; (b) 3D reconstruction of femoral bone; (c) fragment representing the lateral femoral condyle of the femur isolated from the 3D model of the femur.

The femoral bone was measured using 3D scanning (3D GOM Atos Core). The scanning process was preceded by calibration of the instrument and the preliminary preparation of bone surface consisted of cleaning and attaching reference markers (Figure 2(a)). Fifteen scans were performed to digitalize the entire bone, and all individual scans were automatically combined using a process-safe workflow based on a combination of reference points and surface matching. Then, the measured data represented as a point cloud were processed using the GOM Inspect software.

The reconstruction of the 3D bone model (Figure 2(b)) was done by paying special attention to the surface of the lateral condyle of the femur. Noise reduction was performed and all defects in the topological information in the raw 3D model were repaired. In the following steps, the refinement of mesh was performed to smoothen sharp edges, and artefacts on the reconstructed articular surface were removed. The missing parts of the surface, in the form of cavities or gaps, were repaired and finally the virtual object was verified by applying the “leak-tightness inspection” test in the software. The 3D reconstruction of the lateral condyle of the femur (Figure 2(c)) virtually isolated from the 3D representation of whole femoral bone was used as a basis for creating the articular surface of the model of partial RKA endoprosthesis working prototype.

**Figure 3:**
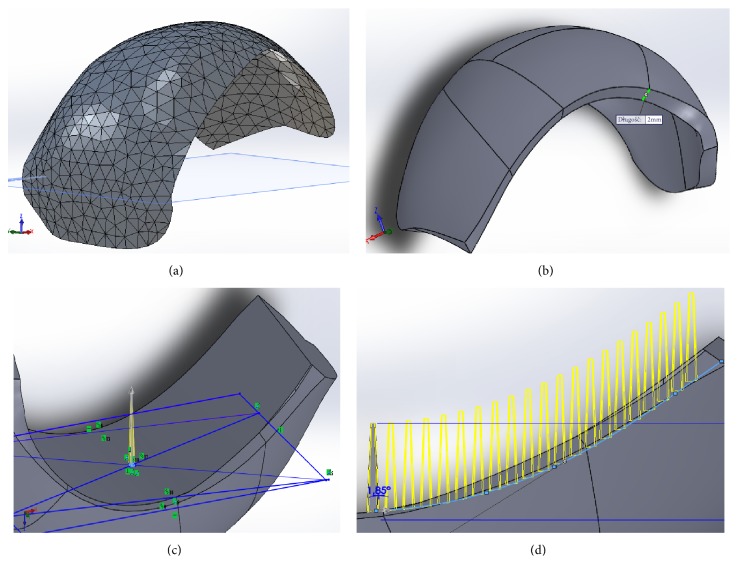
The main stages of CAD-modeling of working prototype of partial RKA endoprosthesis: (a) 3D triangular representation of the articular surface of lateral condyle of the femur as imported to the CAD software; (b) view after adding thickness in the normal direction inwards; (c) a screenshot showing the manner of determining of the initial spike of the MSC-Scaffold; (d) a screenshot showing a preview of the multiplying of spikes using the “curve driven pattern” tool.

In the following step, a 3D triangular representation of the articular surface of the lateral condyle of the femur (Figure 3(a)) was imported into CAD software (Solid Works Premium 2013 x64) using the ScanTo3D plug-in. Then, it was smoothened to level local irregularities and to create a continuous surface, which was thickened by 2 mm (Figure 3(b)) in the normal direction inwards. In the next step, a cylindrical surface was generated under the articular surface of the CAD-modeled working prototype of partial RKA endoprosthesis to create the base for modeling the MSC-Scaffold's spikes.

CAD-modeling of the MSC-Scaffold was initiated by determining the geometric center on the generated cylindrical surface to locate the initial spike. It was determined by the intersection of diagonals of the quadrangle formed on the x-y plane by the combination of the extreme points of the parallel projection of the endoprostheses contour, while the x-y plane was tangential to the cylindrical surface (Figure 3(c)) at this determined center point. The length of the square side of the spike base was established as 0.5 mm and the center of the square was located in the geometric center.

The spikes were designed in the shape of a truncated regular pyramid, with the square on the top having a length equal to 0.25 mm. The total height of the spikes was 4.5 mm. In the subsequent step, the spikes were duplicated using the “curve driven pattern” and “linear pattern” tools (Figure 3(d)). The distance between the spikes bases was 350 *μ*m in both directions. The constructional directives for bioengineering design of the MSC-Scaffold were derived from the results of previous studies by Uklejewski et al. [17–19]. In total, the three variants of the partial RKA endoprosthesis working prototype were generated by creating a series of design types with a ±10% volumetric difference from the base CAD model.

**Figure 4:**
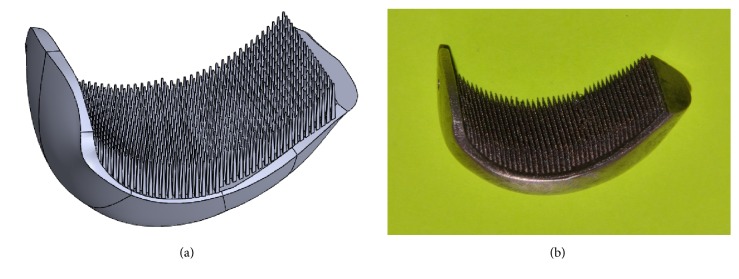
CAD model vs. SLM-manufactured prototype: (a) CAD model of the partial RKA endoprosthesis working prototype with the biomimetic MSC-Scaffold; (b) SLM-manufactured partial RKA endoprosthesis working prototype with the biomimetic MSC-Scaffold.

The final CAD model of the partial RKA endoprosthesis working prototype of the MSC-Scaffold is presented in Figure 4(a). The prototypes were SLM-manufactured on a REALIZER II 250 SLM® machine (MTT Technologies Group, Germany) of Ti6Al4V powder (grain size distribution from 5 to 50 *μ*m). The manufacturing was subcontracted to the Centre of New Materials and Technologies at the West Pomeranian University of Technology, Szczecin, Poland. The applied parameters were laser 100 watts, layer thickness 30 *μ*m, laser spot size 0.2 mm, scan speed 0.4 m/s, and laser energy density 70 J/mm^3^.

The manual postproduction blasting treatment of the MSC-Scaffold was carried out with use of the experimentally customized abrasive mixture composed in equal proportions of white aloxite F220 (±53-75 *μ*m), white aloxite F320 (~29.2 *μ*m ± 1.5%), and microglass blasting beads (~30 *μ*m ± 10%). The SLM-manufactured partial RKA endoprosthesis working prototype is presented in Figure 4(b). The prototypes required subsequent surface finishing, so the grinding and polishing of the articulate surfaces of the resurfacing knee endoprostheses was done.

**Figure 5:**
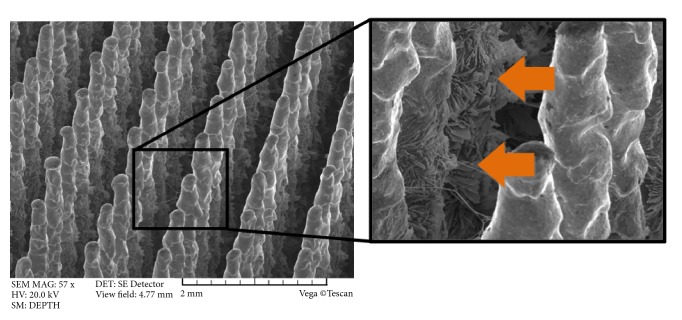
SEM image of the MSC-Scaffold's spikes subjected to electrochemical modification; the arrows show the plate-like shaped hydroxyapatite-like crystals at the lateral surface of the MSC-Scaffold's spikes.

The Ca-P surface modification of bone contacting surfaces of the MSC-Scaffold's spikes was performed with the electrochemical Ca-P cathodic deposition as described in previous work by Uklejewski et al. [26]. In Figure 5 the SEM image of the MSC-Scaffold's spikes subjected to this electrochemical modification is presented. The arrows show the plate-like shaped hydroxyapatite-like crystals at the lateral surface of the MSC-Scaffold's spikes.

The 10 partial RKA endoprosthesis working prototypes were implanted in 10 laboratory swines (breed: Polish Large White). The animals came from a pig breeding being under the supervision of the veterinary department. The surgeries were carried out in a veterinary department operating room with permission of the Local Animal Ethics Committee in Poznan, Poland. The surgery procedure approved by the Local Animal Ethics Committee was painless and stress-free for the animals. anaesthesia was maintained with inhaled isoflurane (Forane) and controlled by pulse oximetry (with a sensor on the swine ear) and heart function monitoring (Drager AT-1) [29, 30].

**Figure 6:**
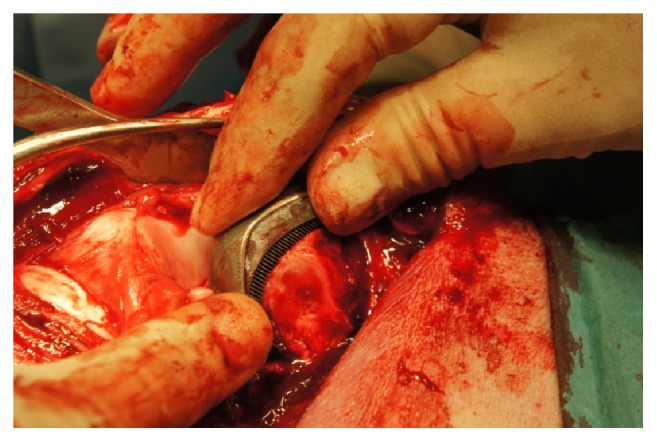
The exemplary partial RKA endoprosthesis working prototype with the MSC-Scaffold implanted into the lateral femoral condyle in swine.

The lateral parapatellar surgical approach was applied to the swine knee joint. No medial approach was used because the small abduction in the hip and the medial localization of the saphenous nerve, the medial saphenous artery and vein [31, 32]. An anterolateral skin incision of ca. 20 cm in length was made over the operated right knee joint. The approach to the knee joint was made between the lateral margin of the patella and the external side of the patellar ligament and then between the vastus lateralis muscle and the rectus femoris muscle. The articular capsule was opened on the lateral side of the patella, and then the patella was dislocated medially. Hemostasis was performed. The patellofemoral region of the knee joint was exposed by osteotomy of the origin of the lateral collateral ligament (LCL) in accordance with the surgical technique to the caudal part of the lateral femoral condyle described by Johnson [33]. The operations were done by a prior bone detachment of the whole femoral part of the lateral collateral ligament of knee joint together with a thin bone fragment. The joint surfaces were prepared using a chisel to ensure proper implant insertion. The implant was inserted with a surgical impactor; the spikes were embedded in the trabecular bone to a depth of around half of the height of the MSC-Scaffold's spikes (Figure 6), allowing limb loading shortly after the resurfacing endoprosthesis implantation. Reattachment of the ligament was done using two trabecular bone screws.

A layered suture was applied to the wound. Antibiotics were introduced after implantation; penicillin powder was administered to the subcutaneous layer at the end of surgery, then the wound was covered by a mesh impregnated with penicillin, and an antiseptic dressing was applied. After the surgery, amikacin (Biodacyna) was given at 1 g twice a day i.v. (or i.m.) for 3 days. The animals were kept in the veterinary department to achieve full mobility and functional welfare in the breeding rooms. Husbandry conditions including the type of food met the standard procedures approved by the Local Animal Ethics Committee. On the third day after surgery, the swine were allowed to attempt full weight bearing. A four-week postoperative radiological examination was performed in the veterinary department with premedication of the animals as during implantation, using the Stenoscop Plus Mobile C-Arm X-ray apparatus (GE Medical Systems, Japan).

Clinical assessment of joint stability after partial RKA implantation has been adapted from [28] with modification: medial/lateral stability of joint measured in full extension. Radiological assessment of stability (loosening of component) was evaluated in 4-grade scale. Radiolucency around the component was evaluated in terms of thickness and positioning [27].

Eight weeks after implantation, the explantation was performed in the veterinary department operating room (premedication and general anaesthesia as done for implantation), and the knee joints with the RKA endoprosthesis working prototypes were harvested from animals; the procedure was followed by euthanasia of the animals (Morbitan/pentobarbital natrium at a lethal dose of 200 mg/kg BW i.v., according to the protocol approved by the Local Animal Ethics Committee). The specimens of operated swine knee joint with the implanted working prototypes of partial RKA endoprosthesis resected 8 weeks after implantation were radiologically examined using the XPERT®40 Digital Specimen Radiography System (Kubtec, USA) and immersed in 10% neutral buffered formalin for seven days.

**Figure 7:**
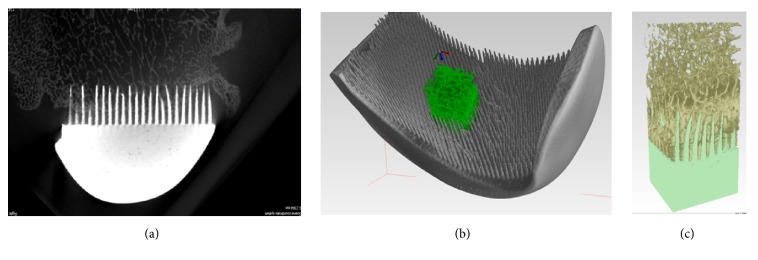
The main stages of micro-CT reconstruction of explanted knee joint: (a) exemplary 2D slice of the micro-CT reconstructed knee joint specimen with the RKA endoprosthesis working prototype with the MSC-Scaffold; (b) 3D view of the bone-implant specimen (RKA endoprosthesis working prototype) with (c) an exemplary fragment of the bone-implant specimen extracted for subsequent qualitative analysis.

The micro-CT examination of the retrieved knee joints was conducted using a high-energy micro-CT scanner (SkyScan 1173 micro-CT scanner, Bruker, Belgium). During the micro-CT image acquisitions (carried out during the first day of the fixation process) the specimens remaining immersed in 10% formalin were mounted on a rotary stage and scanned in their entirety. The scanning parameters were source voltage 130 keV, source current 61 *μ*A, resolution 9.92 *μ*m, filter 0.25 mm brass, exposure time 4000 ms, rotation 360°, every 0.2°, with a scanning time of about 6h. 3D visualization and 2D image analysis of micro-CT reconstructed knee joints with the MSC-Scaffold were performed using the SkyScan CT analyzer. In the bone-implant 3D reconstructions, the four cuboidal fragments were extracted for subsequent qualitative and quantitative analyses. In the extracted regions of the bone-implant specimens, elements such as the Ti-alloy of the MSC-Scaffold, trabeculae, and soft tissues including bone marrow were identified on the basis of radiological density. In each extracted region of the bone-implant specimens, the six reference levels of area perpendicularly intersecting the axes of the MSC-Scaffold's spikes were established below their tops and spaced 0.5 mm from each other. The proportions of Ti-alloy MSC-Scaffold, trabeculae, and soft tissues were measured in the specimen reconstructions. In Figure 7(a) an exemplary 2D slice of a micro-CT reconstructed knee joint with the RKA endoprosthesis working prototype with the MSC-Scaffold is shown, while in Figure 7(b), a 3D view of a bone-implant specimen with an exemplary cuboidal fragment extracted from the bone-implant 3D micro-CT reconstruction is presented.

Following micro-CT examination, the specimen fixation in 10% formalin solution was continued up to 7 days, followed by dehydration in serial concentrations of ethanol (70%, 80%, 90%, 99%, 100%, v/v) for 2 days at each concentration, and then the specimens were embedded in resin. Thick sections (200 *μ*m) were cut using a rotating wheel saw (IsoMet 4000 Linear Precision Saw, Buehler, Germany) and ground to sections with a thickness of 20 *μ*m using the MetaServ 250 grinder-polisher (Buehler, Germany). Thin sections (20 *μ*m) were stained with hematoxylin-eosin and examined by light microscopy (Olympus CX41, Olympus, Japan).

The analysis used Statistica 12.0 (StatSoft) software. Descriptive values of variables are expressed as means ± standard deviation or medians (minimum-maximum).

## 3. Results and Discussion

**Figure 8:**
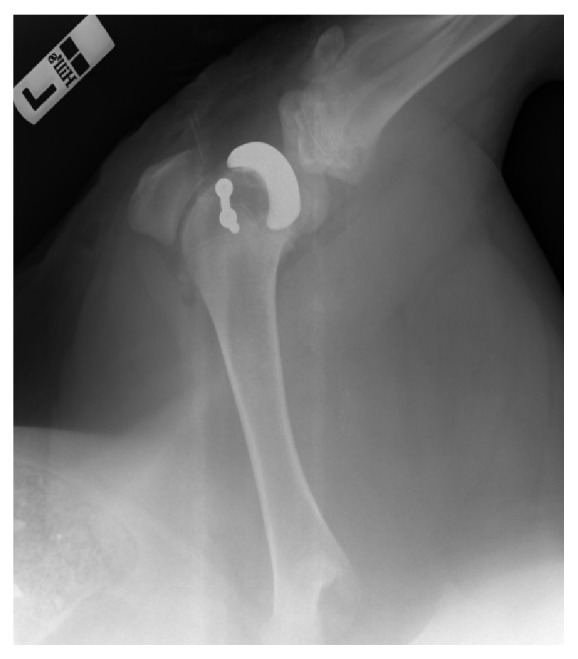
The exemplary radiogram of operated swine knee joint at 4 weeks after implantation.

**Figure 9:**
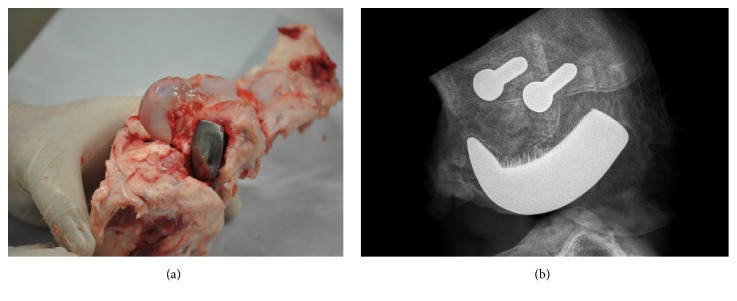
The retrieved specimen vs. its X-ray radiogram: (a) the exemplary specimen of operated swine knee joint with the implanted working prototype of partial knee resurfacing endoprosthesis harvested at 8 weeks after implantation; (b) the exemplary 2D digital X-ray radiogram of the resected at 8 weeks after implantation swine knee joint showing spaces between the spikes of the MSC-Scaffold of the implanted RA endoprosthesis working prototype penetrated with bone tissue and the two trabecular bone screws used for the reattachment of the femoral part of the lateral collateral ligament of the swine knee joint.

The exemplary result of the radiological examination of operated swine knee joint at 4 weeks after implantation is presented in Figure 8.  Figure 9(a) shows the exemplary specimen of operated swine knee joint with the implanted working prototype of partial RKA endoprosthesis resected surgically at 8 weeks after implantation.  Figure 9(b) shows the exemplary X-ray radiogram of the resected swine knee joint.

A summary of the data dealing with investigations on RHA endoprosthesis working prototype implanted in 10 swines is presented in Table 1.

The operative procedure facilitated the approach to the entire femoral condyle for partial RKA. It allowed for good fixation of the endoprosthesis. In nine cases good (30%) or very good stability (60%) of the operated knee joints was observed and no implant migration, with the exception of one case, where aseptic complication occurred (Table 2). The clinical and radiological examination confirmed very good stability of the knee joints (radiolucency less than 2 mm). In the remaining nine animals, no destruction of the femoral condyle was found in the harvested bone-implant specimens 8 weeks after implantation. The integrity of the LCL was done by holding the limb in extension and performing a varus stress test. Using one hand to stabilize the femur, and the other to hold the end of the tibia, it was applied an inward force to the joint. If the lateral collateral ligament is torn, an “opening” of the joint is apparent; stability in the clinical examination was 4 on the 5-point Likert scale (Table 3). The mean weight of the animals was 82.0 ± 7.6 kg (Table 3). The X-ray radiograms of all the resected swine knee joints showed that the spaces between the MSC-Scaffold's spikes were penetrated with bone tissue.

**Figure 10:**
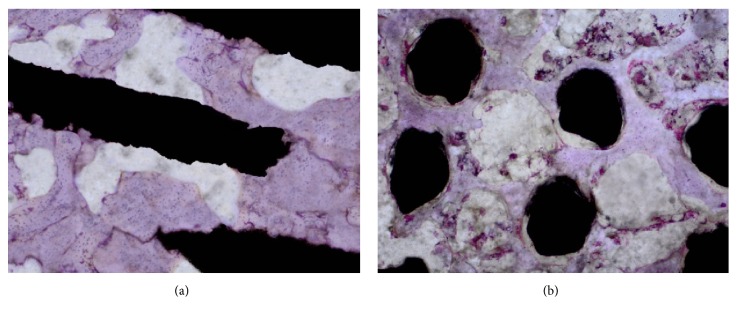
The exemplary 8th week after surgery histological sections (H+E staining) showing the interspike pore space of the MSC-Scaffold penetrated by matured trabecular bone tissue: bone trabeculae of periscaffold bone are considered as in equal age; bone-implant sections in (a) the longitudinal and (b) crosswise directions to the axis of the spikes.

Figure 10 presents the exemplary 8th week after surgery histological sections in the longitudinal (Figure 10(a)) and crosswise (Figure 10(b)) directions to the spikes axis. No morphological inflammatory exponents were observed in the 8th week after the surgery histological sections of periscaffold bone tissue. In the 8th week after the surgery, almost all the MSC-Scaffold's spikes in longitudinal and transversal sections are surrounded by mature bone tissue. No morphological exponents of osteogenesis process were found. Periscaffold trabecular bone in these histological sections is considered as of equal age and mature.

**Figure 11:**
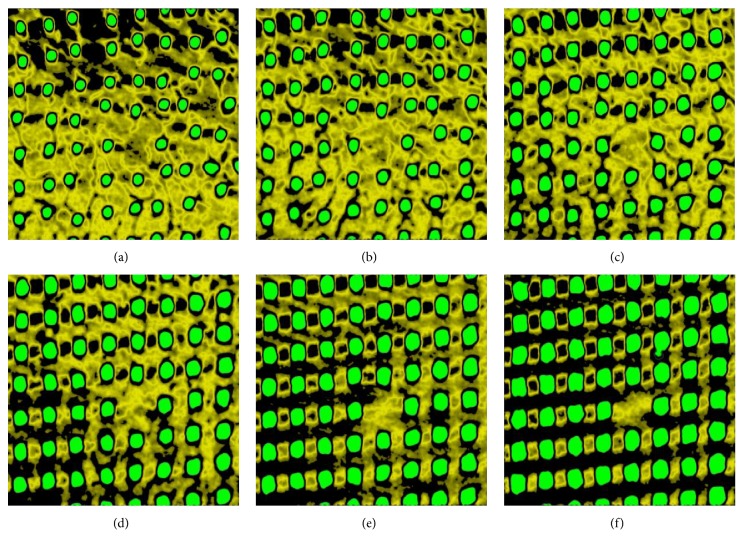
Series of six exemplary micro-CT scan slices of the explanted bone-implant specimen established at six reference levels at the distances of (a) 3.5 mm, (b) 3.0 mm, (c) 2.5 mm, (d) 2.0 mm, (e) 1.5 mm, and (f) 1.0 mm from the spike bases of the MSC-Scaffold.

In Figure 11, a series of six images representing the six reference levels of the micro-CT scan slices of the explanted bone-implant specimen is presented. On the basis of radiological density, three elements including the Ti-alloy MSC-Scaffold, trabeculae, and soft tissues are identified in green, yellow, and black, respectively.

**Figure 12:**
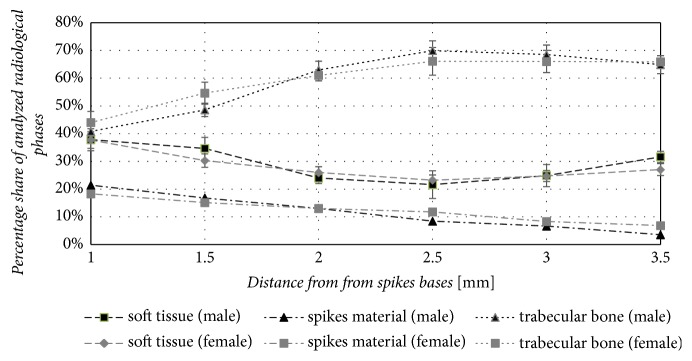
The percentage share of analyzed radiological phases: trabecular bone, spike material, and soft tissue of the explanted bone-implant specimen as a function of the distance from the spike bases.

The qualitative analysis of the microstructure of cancellous bone radiological phase in the interspike space of the MSC-Scaffold reveals—in the reference levels closer to the spikes tops (Figures 11(a)–11(c))—the trabecular structure similar to the cancellous bone neighbouring the MSC-Scaffold. In the micro-CT slices representing the reference levels closer to the bases of the spikes (Figures 11(d)–11(f)) there is observed the initial stage of bone tissue bone ingrowth in the form of creeping ingrowth of bone tissue on the lateral surfaces of the spikes; this initial stage was followed by the appositional growth of bone tissue filling the interspike spaces of the MSC-Scaffold. The results of the quantitative identification of bone-implant integration in the region of the MSC-Scaffold's spikes are presented in Figure 12, showing the changes in the proportions of each identified radiological phases as a function of the distance from the spikes bases.

The share of cancellous bone increased with the distance from the spikes bases from 44 ± 4% (female) and 41 ± 4% (male), to a maximum value of 66 ± 5% (female) and 70 ± 4% (male) at a distance of 2.5 to 3.0 mm from the spikes bases, while the share of soft tissue decreased from approximately the same share of 38 ± 4% for both sexes to a minimum value of 23%  ± 5% (female) and 22%  ± 4% (male) at a distance of 2.5 mm from the spikes bases. The share of Ti-alloy MSC-Scaffold spikes material decreased linearly with the distance from the spikes bases, consistent with the mathematical expectation arising from the geometrical construction of the spikes; this confirms the reliability of all obtained results.

The distance from the spikes bases (2.5 to 3.0 mm) at which the share of cancellous bone reached the maximum corresponds to the initial embedding depth of the MSC-Scaffold performed by the operating surgeon. In this region, most of the mechanical loading is transferred from the MSC-Scaffold to the surrounding trabecular bone, which causes bone remodeling in the highest share of remodeled cancellous bone in relation to soft tissues.

Components of all the contemporary partial knee resurfacing endoprostheses (e.g., iBalance® unicompartmental knee arthroplasty, MAKOplasty® partial knee resurfacing, Oxford® partial knee resurfacing, etc.) are fixed in bone with cement and stabilizing pegs or short stems. Proposed by us the prototype MSC-Scaffold for RKA, applicable also for the most articular joints (hip, shoulder, elbow, ankle, hand, and foot joints), allows for the entirely cementless and biomimetic—the MSC-Scaffold spikes mimic the interdigitations of subchondral bone—fixation of components the periarticular trabecular bone and to allow patient for full weight bearing shortly after the surgery. This prototype construction solution has international patents [20–22]. In the current literature, there is no construction solution of resurfacing endoprosthesis allowing for the entirely cementless and biomimetic fixation of its components.

To prove that the biomimetic fixation for resurfacing arthroplasty by means of the innovative MSC-Scaffold allows for the entirely cementless and biomimetic fixation of the components of RA endoprostheses in the periarticular cancellous bone, the partial RKA endoprosthesis working prototype with the MSC-Scaffold was designed for swine using reverse engineering methods. The external shape of knee endoprosthesis was designed for a swine of mean weight of 82.0 ± 7.6 kg, whereas the MSC-Scaffold for the endoprosthesis was designed according to the assumptions of the patent [20–22] with taking into account the technological considerations [34] and biological restricts regarding the expected bone tissue ingrowth [35–39]. To design of the MSC-Scaffold microstructure, the background regarding bone ingrowth in case of porous coatings [40–43] and three-dimensionally printed porous metal scaffolds [44] was followed. Since the original MSC-Scaffold differs from the above porous structures, the biomimetics in terms of the spikes' size adjustment to the intertrabecular space of the periarticular bone [45] and the spikes arrangement conditioning structural accessibility for ingrowing bone tissue was assessed by the approach proposed in [23]. The constructional features of the MSC-Scaffold allow for its unit loadability ca. 2.5 N/mm^2^ with the scaffold spikes embedded in the bone to the half high of the spikes. Such the unit loadability of the MSC-Scaffold corresponds to the middle zone of the elastic range of the stress-strain characteristics of periarticular cancellous bone [24].

The SLM-manufactured partial RKA endoprosthesis working prototypes with the MSC-Scaffold after Ca-P surface modification of bone contacting surfaces of the MSC-Scaffold were implanted in 10 swines (breed: Polish Large White). The selection of an adequate animal model for implantation of an innovative surface prosthesis is very important for transferring the results of experimental studies to clinical applications [46–48]. Of the species of large animals, the dog probably has the most similar bone structure to humans; however, ethical considerations restrict the use of companion animals for medical research.

So far, the swine model successfully used in studies of bone defects. Similar density, anatomy and bone microstructure to the human bone were confirmed. The limitation in the studies turned out to be a large mass of animals and accelerated bone growth makes it difficult the differentiation of early and late remodeling [47–49]. Direct reference to pig implant testing may also be difficult due to the shorter length of the tibia and femur bone than in human [47–49]. The swines have well-developed Havers systems in a growing and mature bone [47]. In pigs blood circulation, metabolism and bone remodeling largely correspond to these processes in humans [50]. Construction of synovial joint is structurally cartilage and ligament system very similar to a human joint [48]. Therefore, the swine model appears to be an animal of choice in the case of surgical implantation [50–52].

Of the 10 swines used in the study, there were 5 female and 5 male. The group of animals used in the study was fairly uniform in terms of age (mean: 8.2 ± 0.8 months) and body weight (mean: 82 ± 7,6 kg). The animals were healthy, in a good general condition; none of the animals were sterilized. It can be considered that their physiological development and hormonal status were correct. The weight gain of animals used in the experiment observed at 8th week after surgery was about 10-15% – this means that the unit loadability of the MSC-Scaffold was still in the elastic range of the stress-strain curve of periarticular cancellous bone, as required.

The sex-related differences in bone physiology may correspond to differences in bone ingrowth into the porous coatings of implants. High-dose estrogen has been shown to stimulate the differentiation and activity of osteoblasts in vitro and increase bone formation in animal models, and also in postmenopausal women [53–55]. Shih et al. [56] evaluated the effects of sex and estrogen therapy on bone ingrowth into porous coated implants in an animal model (dogs). Three months after implantation, histological examination showed significantly more bone ingrowth in areas of porous coating with cortical bone contact than in areas with cancellous bone contact (pore spaces of investigated porous coating allowed formation in them of osteons of cortical bone, whereas the ingrowth of cancellous bone trabeculae in the porous coating was restricted). Bone ingrowth was essentially the same in male and female dogs. Ovariectomized dogs showed less overall bone ingrowth than male and female control dogs; short-term high-dose estradiol treatment did not increase bone ingrowth volume fraction. The authors concluded that the kind of bone contact is the key factor affecting the bone ingrowth into the porous coating of the implant [56].

In the above context, the study of the influence of gender on the adaptation of the scaffold in the investigated group of pigs during the 8-week period of postoperative follow-up did not make much sense and did not fall within the planned research. The main type of adaptation of the bone tissue to the scaffold that occurred during the study was the adaptation of peri-implant bone tissue to its elastic deformations and stresses induced by mechanical loading of the implanted knee joint resurfacing endoprosthesis with the MSC-Scaffold during daily animal activity. The ingrowth of the bone tissue into the scaffold, observed in micro-CT imaging, occurred similarly, regardless of pigs gender (Figure 12).

Many hip and knee arthroplasties are performed in aged patients being often in osteoporotic status due to menopause and/or aging; in such cases, most orthopedists prefer the use of cemented endoprostheses. Treatment with resurfacing arthroplasty usually requires the nonosteoporotic periarticular cancellous bone. Therefore, the study of the influence of animal age on the results obtained was not within the scope of the planned research.

Our prototype of biomimetic MSC-Scaffold can be regarded as a promising breakthrough in bone-implant advanced interfacing in joint resurfacing endoprostheses fixation technique. This advanced biomimetic Ti-alloy prototype interfacing with bone manufactured owing to the advanced laser technology, opens a new generation of the first biomimetic RA endoprostheses, which can be applied for most diarthrodial joint arthroplasties (hip, knee, shoulder, elbow, etc.) used in orthopaedic surgical treatment.

## 4. Conclusions

The clinical, radiological, histological, and micro-CT examinations on the biomimetic fixation of the partial RKA endoprosthesis working prototype in 10 swines showed that

- a scaffolding effect was obtained with the MSC-Scaffold of the RKA endoprosthesis working prototypes, i.e., the interspike pore space was penetrated by newly formed bone tissue, providing primary biological fixation of all implanted prototypes in periarticular cancellous bone;
- in deeper regions, near to the spikes bases of the MSC-Scaffold, an early stage of bone-implant osseointegration in the form of creeping ingrowth of bone tissue on the lateral surfaces of the spikes was observed in the micro-CT reconstruction of the explanted bone-implant specimens;
- the micro-CT results showed the highest percentage of bone tissue ingrowths into the MSC-Scaffold at a distance of 2.5÷3.0 mm from the spikes bases.

The results show that the biomimetic fixation for resurfacing arthroplasty by means of the innovative MSC-Scaffold allows for the entirely cementless and biomimetic fixation of the components of RA endoprostheses in the periarticular cancellous bone with very good clinical stability.

## Figures and Tables

**Table 1:** Summary of data dealing with investigations on swines with implanted partial RKA endoprosthesis working prototype.

No of swine	Weight (kg)	Sex	Age (months)	Knee	Stability in radiological examination	Stability of operated joint in clinical examination	Destruction of femoral condyle
					(based on [27])	(Likert scale)	
						(based on [28])	
1	76	M	8	left	good	4	none
2	91	F	9	right	good	4	none
3	77	F	7	left	very good	5	none
4	69	M	8	left	very good	5	none
5	94	F	9	right	migration	2	none
6	78	M	8	left	very good	5	none
7	91	F	9	left	good	3	none
8	86	M	8	right	very good	4	none
9	81	M	9	right	very good	5	none
10	74	F	7	right	very good	4	none

**Table 2:** Characteristics of stability of operated swine knee joints in the radiological examination in 10 swines with an implanted RKA endoprosthesis working prototype (N=10).

Stability in radiological examination	Number (percentage)
migration	1 (10%)
good	3 (30%)
very good	6 (60%)

**Table 3:** Characteristics of weight and stability in the clinical examination of 10 swines with implanted RKA endoprosthesis working prototype.

Parameters	Mean ± SD	Median (min-max)
Weight (kg)	82.0 ± 7.6	80.5 (69-94)
Age (months)	8.2 ± 0.8	
Stability scale in clinical examination		4 (2-5)

## Data Availability

The data used to support the findings of this study are available from the corresponding author upon request.
